# Investigation of Nasal/Oropharyngeal Microbial Community of COVID-19 Patients by 16S rDNA Sequencing

**DOI:** 10.3390/ijerph18042174

**Published:** 2021-02-23

**Authors:** Martina Rueca, Andrea Fontana, Barbara Bartolini, Pierluca Piselli, Antonio Mazzarelli, Massimiliano Copetti, Elena Binda, Francesco Perri, Cesare Ernesto Maria Gruber, Emanuele Nicastri, Luisa Marchioni, Giuseppe Ippolito, Maria Rosaria Capobianchi, Antonino Di Caro, Valerio Pazienza

**Affiliations:** 1 National Institute for Infectious Diseases “L. Spallanzani”, IRCCS, 00149 Rome, Italy; martina.rueca@inmi.it (M.R.); barbara.bartolini@inmi.it (B.B.); pierluca.piselli@inmi.it (P.P.); antonio.mazzarelli@inmi.it (A.M.); cesare.gruber@inmi.it (C.E.M.G.); emanuele.nicastri@inmi.it (E.N.); luisa.marchioni@inmi.it (L.M.); giuseppe.ippolito@inmi.it (G.I.); maria.capobianchi@inmi.it (M.R.C.); 2 Biostatistics Unit Fondazione-IRCCS “Casa Sollievo della Sofferenza” Hospital, 71013 San Giovanni Rotondo , Italy; a.fontana@operapadrepio.it (A.F.); m.copetti@operapadrepio.it (M.C.); 3 Cancer Stem Cell Unit, ISBReMIT Fondazione-IRCCS “Casa Sollievo della Sofferenza” Hospital, 71013 San Giovanni Rotondo, Italy; e.binda@operapadrepio.it; 4 Gastroenterology Unit Fondazione-IRCCS “Casa Sollievo della Sofferenza” Hospital, 71013 San Giovanni Rotondo, Italy; f.perri@operapadrepio.it

**Keywords:** microbiota, SARS-CoV2, Nasal/Oropharyngeal

## Abstract

Since December 2019, SARS-CoV-2 infection has been still rapidly spreading, resulting in a pandemic, followed by an increasing number of cases in countries throughout the world. The severity of the disease depends on the patient’s overall medical condition but no appropriate markers are available to establish the prognosis of the patients. We performed a 16S rRNA gene sequencing, revealing an altered composition of the nasal/oropharyngeal (NOP) microbiota in 21 patients affected by COVID-19, paucisymptomatic or in an Intensive Care Unit (ICU), as compared to 10 controls negative for COVID-19 or eight affected by a different Human Coronavirus (HKU, NL63 and OC43). A significant decrease in Chao1 index was observed when patients affected by COVID-19 (in ICU) were compared to paucisymptomatic. Furthermore, patients who were in ICU, paucisymptomatic or affected by other Coronaviruses all displayed a decrease in the Chao1 index when compared to controls, while Shannon index significantly decreased only in patients under ICU as compared to controls and paucisymptomatic patients. At the phylum level, Deinococcus-Thermus was present only in controls as compared to SARS-CoV-2 patients admitted to ICU, paucisymptomatic or affected by other coronaviruses. Candidatus Saccharibacteria (formerly known as TM7) was strongly increased in negative controls and SARS-CoV-2 paucisymptomatic patients as compared to SARS-CoV-2 ICU patients. Other modifications were observed at a lower taxonomy level. Complete depletion of Bifidobacterium and Clostridium was exclusively observed in ICU SARS-CoV-2 patients, which was the only group characterized by the presence of Salmonella, Scardovia, Serratia and Pectobacteriaceae. In conclusion, our preliminary results showed that nasal/oropharyngeal microbiota profiles of patients affected with SARS-CoV-2 may provide valuable information in order to facilitate the stratification of patients and may open the way to new interventional strategies in order to ameliorate the outcome of the patients.

## 1. Introduction

At the moment of writing, more than 111.7 million confirmed cases of COVID-19 are reported worldwide, with 2.4 million deaths recorded (World Health Organization (WHO) update: https://covid19.who.int/ accessed on 22 February 2021). A novel coronavirus classified as severe acute respiratory syndrome coronavirus-2 (SARS-CoV-2) was identified as the cause of acute respiratory disease in the city of Wuhan in the Hubei Province of China, which is still rapidly spreading, resulting in a pandemic and an increasing number of cases in countries throughout the world [1]. Although scientists agree about the main transmission route of this novel virus, consisting mainly of respiratory droplets and close contact, multiple issues have emerged in the management of COVID-19. While the most common symptoms in mild to moderate symptomatic patients are fever and dry cough, followed by other symptoms such as fatigue, headache, myalgia and arthralgia, and digestive symptoms such as nausea, diarrhea and belly pain, [2,3] the full spectrum of clinical manifestations associated with COVID-19 in humans remains to be determined [4]. As the disease progresses, a series of complications tend to develop, especially in the elderly or in patients with comorbidities. Although several ongoing clinical trials are investigating the efficacy of some promising antivirals, ozone therapy, convalescent plasma transfusion, biologics and anti-rheumatoid arthritis drugs, to date, no specific treatment has been proven for COVID-19 [5]. Since data are limited, there is an urgent need for fast-track research, especially to distinguish patients with a severe clinical outcome from asymptomatic patients who do not display any particular evident sign of infection [6]. Recent studies have shown that the host’s microbiota can positively or negatively influence the course of a wide range of infections [7]. With their trillion microorganisms, the host’s microbiota are known to play a major role in several vital functions, spanning from metabolic functions to immune system activities. Most of them reside within the gastrointestinal tract but they also colonize the skin, mammary glands, uterus, ovarian follicles, lung, saliva, oral cavity and conjunctiva, among others. In all these sites, the commensal microbial flora shape the local immune system in order to maintain a “healthy homeostasis” between the host and the microbes [8]. The upper respiratory tract consists of the major site of entry and infection by SARS-CoV-2 and the human nasal mucosa harbors a community of microorganisms that plays a key role not only in mucosal homeostasis but also in resistance to infection [9]. It has been reported that the human nose microbiota, including bacteria of the genus Lactobacillus, provide their function for the respiratory tract [10]. Furthermore, a number of studies have shown that nasal microbiome composition can directly influence the progression of acute respiratory tract infections, which are caused, in most cases, by viruses [11]. Conversely, the role of the upper airway microbiota in SARS-CoV-2-infected patients is not yet completely understood, even though it is now clear that the nasal barrier is the first defensive line to avoid infection. It is believed that commensal bacteria residing within the nasal cavity can protect from opportunistic pathogens by selective inhibition or by producing toxic molecules until territorial or metabolic niche establishment [12]. Microbiota manipulation is an approach already exploited in different medical fields, from cancer to metabolic disorders and virus infection [13,14,15,16]. The literature suggests different mechanisms by which the viruses could use bacteria and their products to counterattack environmental adversities and dispatch their function, crossing the host cell barriers [17]. The host’s microbiota can either regulate or can be, in turn, disrupted by invading viruses with the consequence of suppressing or stimulating viral infections [16]. The respiratory system is the main place of replication of SARS-CoV-2 and, to date, no data are available regarding the link between the upper respiratory tract microbiota and COVID-19 infection and how this interaction could have an impact on clinical outcome. In our pilot study, we analyzed the 16S nasal/oropharyngeal (NOP) microbiota profile in different groups of COVID-19 patients, those affected with other coronaviruses, and healthy controls.

## 2. Materials and Methods

### 2.1. Study Population

A total of 39 subjects were recruited for this study from the end of February 2020 to the beginning of May 2020, of which there were 21 patients with a laboratory-confirmed infection by SARS-CoV-2 at National Institute for Infectious Diseases “L. Spallanzani”, IRCCS, Rome, Italy, and were divided as follows: 10 patients were admitted to the Intensive Care Unit (SARS-CoV-2 ICU) and 11 were paucisymptomatic presenting mild (weakness or headache) to moderate (fever or cough) COVID-19 symptoms (SARS-CoV-2 Pauci), not requiring admission to ICU. Furthermore, ten healthy subjects (Neg Controls) and eight subjects affected by human coronaviruses (HCoVs) 5 HKU1, 2 NL63 and 1 OC43 (Other HCoVs) were enrolled in this study. Regarding the ICU patients, six of them died (five during hospitalization and one after discharge from the hospital) and were characterized by the presence of different comorbidities such as 1 heart disease, 1 liver disease, 1 major depression, 1 cancer, 1 dyslipidemia and 1 Parkinson disease.

### 2.2. Virus Detection

A 350 µL aliquot of a nasal/oropharyngeal swab was used for nucleic acid extraction using the QIAsymphony automatic extractor with DSP Virus/Pathogen Midi Kit (QIAGEN, Hilden, Germany) following the manufacturer’s protocol. RT-PCR was used to detect SARS-Cov-2 and a multiplex RT-PCR including other HCoVs was used for differential diagnosis of respiratory pathogens.

### 2.3. Sample Collection, DNA Extraction and Next-Generation Sequencing of Bacterial 16S Ribosomal RNA Gene

Nasal/oropharyngeal swabs were collected from patients and Microbial DNA was extracted from 350 µL of samples using the QIAsymphony automatic extractor with DSP Virus/Pathogen Midi Kit (QIAGEN, Hilden, Germany) according to the manufacturer’s protocol. 16S gene hypervariable regions were amplified using two separate pools of primers targeting V2–4–8 and V3–6 and 7–9 regions with the Ion 16S Metagenomics Kit (ThermoFisher, Milan; Italy). Amplification was performed separately for the two pools of primer for each sample, then the two reactions tubes were pooled and used for library preparation in the following steps. The Ion Xpress Plus Fragment Library Kit (ThermoFisher, Milan; Italy) was used following the manufacturer’s instructions. Produced libraries were then purified using the AMPure XP Beads (Beckman Coulter, Milan; Italy) and quantified using the Ion Library TaqMan Quantitation Kit (ThermoFisher, Milan; Italy) on the 7900 instrument (Applied Biosystem) and the High-Sensitivity DNA Kit on Bioanalyzer 3100 (Agilent, Santa Clara (CA) United States). Sequencing was performed on an Ion 530 chip using the Ion S5 Sequencer (ThermoFisher, Milan; Italy) obtaining, on average, 5 × 10^5^ reads per sample (range: 3 × 10^5^–9 × 10^5^).

### 2.4. Bioinformatic Analysis

Demultiplexed FASTQ files were analyzed as previously reported [18] performing the following pipeline: read pairs are controlled at quality level (i.e., trimming, clipping and adapter removal), based on FastQC and BBDuk, and are mapped with BWA-MEM against the database (based on NCBI) using the 16S Metagenomics GAIA 2.0 software (http://www.metagenomics.cloud accessed on 13 October 2020, Sequentia Biotech 2017, Barcelona, Spain). Differential expression analysis using a DESeq2 package was conducted to test for differential analysis by use of negative binomial generalized linear models. Only changes with FDR below 0.05 were considered significant. The percent similarities used to determine species and genus calls were 93% at genus level and 97% at species level. Chao1 and Shannon indexes were also calculated by GAIA Sequentia Biotech software. Principal Coordinate Analysis (PCoA)was obtained with GAIA software based on Bray–Curtis dissimilarities.

### 2.5. Statistical Analysis

Clinical characteristics of the study population were reported as median along with interquartile range (IQR, i.e., first-third quartiles) and observed frequencies (and percentages) for continuous and categorical variables, respectively. Comparisons among groups were performed using the nonparametric Kruskal–Wallis and Dwass–Steel–Critchlow–Fligner tests to assess both the presence of overall and pairwise differences, respectively. Furthermore, boxplots were performed to depict the distribution of the age at recruitment and both richness and diversity indices (i.e., Chao1 and Shannon) among all subjects’ groups, highlighting all statistically significant pairwise differences. In order to cluster the microbial communities at the genus operational taxonomic unit (OTU) level, PCoA was performed using the Bray–Curtis dissimilarity matrix, and the corresponding multidimensional scaling (MDS) plot was produced with respect to the first two eigenvectors (axes 1 and 2). Two-sided *p*-values < 0.05 were considered for statistical significance. All statistical analyses were performed by the computing environment R (R Development Core Team 2008, version 4.0, Vienna, Austria), packages: table one, NSM3). Plots were produced using Prism version 8, (GraphPad; San Diego, CA, USA). Venn diagrams were obtained with Venny 2.1.0 (National Center of Biology; Madrid; Spain).

## 3. Results

### 3.1. Study Population

**Figure 1:**
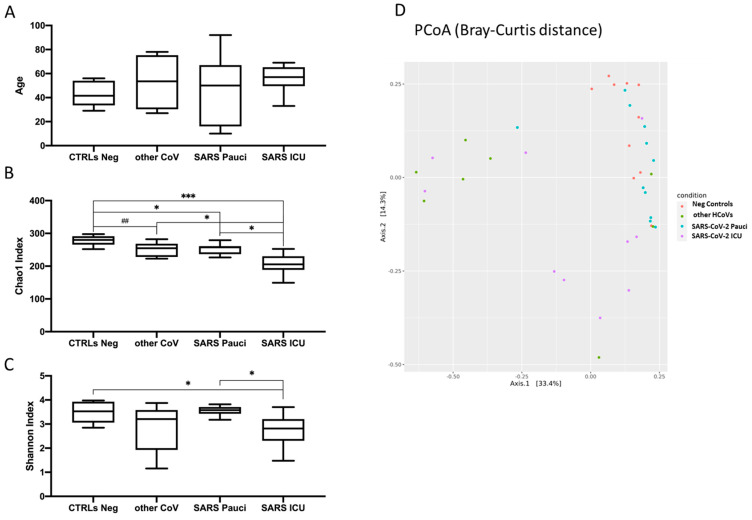
Boxplots of age (**A**), Chao1 index of species richness (**B**) and Shannon index of species diversity (**C**) in healthy subjects (Neg Controls), patients infected with other human coronaviruses (Other HCoVs), paucisymptomatic patients (SARS-CoV-2 Pauci) and patients in Intensive Care Units (SARS-CoV-2 ICU). (## = *p* < 0.10; * = *p* < 0.05; ** = *p* < 0.01; *** = *p* < 0.005). (**D**) Multidimensional Scaling plot from Principal Coordinates Analysis (PCoA) based on Bray–Curtis distances at genus level showing a clustering pattern among samples obtained from Neg controls (red), other HCoVs (green), SARS-CoV-2 Pauci (blue) and SARS-CoV-2 ICU (violet).

The study population included 39 patients: 10 SARS-CoV-2 ICU, 11 SARS-CoV-2 Pauci, 10 Neg Controls and 8 Other HCoVs. The median age was 57 (IQR: 52–64), 50 (IQR: 22–65), 53.5 (IQR: 30–71), and 41.5 (IQR: 34–52) years for SARS-CoV-2 ICU, SARS-CoV-2 Pauci, Other HCoVs and Neg Control groups, respectively; (Figure 1A). Percentages of male patients were 60% and 75% for SARS-CoV-2 ICU and Other HCoVs, respectively, and 27.3% and 30% for SARS-CoV-2 Pauci and Neg Controls, respectively.

### 3.2. Microbial Richness and Diversity Indices in SARS-CoV-2 and Other HCoVs Patients as Compared to Neg Controls

As a first step, we tested whether our three different groups of patients displayed a statistically significant difference among them, as age is one of the key factors influencing the microbiota profiles. As shown in Figure 1A, no differences were observed among the three different groups. We then evaluated the richness and Shannon indices among the different groups in order to understand the nasal/oropharyngeal microbiota alterations between SARS-CoV-2 Pauci patients, SARS-CoV-2 ICU patients and Other HCoVs patients as compared to Neg Controls for SARS-CoV-2. As shown in Figure 1B, the Chao1 index resulted in significantly decreased SARS-CoV-2 ICU as compared to SARS-CoV-2 Pauci patients (*p* = 0.03), Other HCoVs (*p* = 0.02) and Neg Controls (*p* = 0.001). Although the nasal/oropharyngeal microbial richness was decreased in all three groups as compared to controls, SARS-CoV-2 ICU patients displayed the lowest median value. The same trend was also observed for Shannon index in only SARS-CoV-2 ICU patients displaying a statistically significant decrease as compared to Neg Controls and SARS-CoV-2 Pauci patients (*p* = 0.04 and *p* = 0.03, respectively (Figure 1C). The PCoA based on the Bray–Curtis dissimilarity was shown in Figure 1D (MDS plot) and displayed distinct patterns among the three groups of Neg Controls (red), Other HCoVs (green), SARS-CoV-2 Pauci patients (blue) and SARS-CoV-2 ICU unit (violet).

### 3.3. Nasal/Oropharyngeal Microbiota in SARS-CoV-2 Patients and Other HCoVs as Compared to Controls

**Figure 2:**
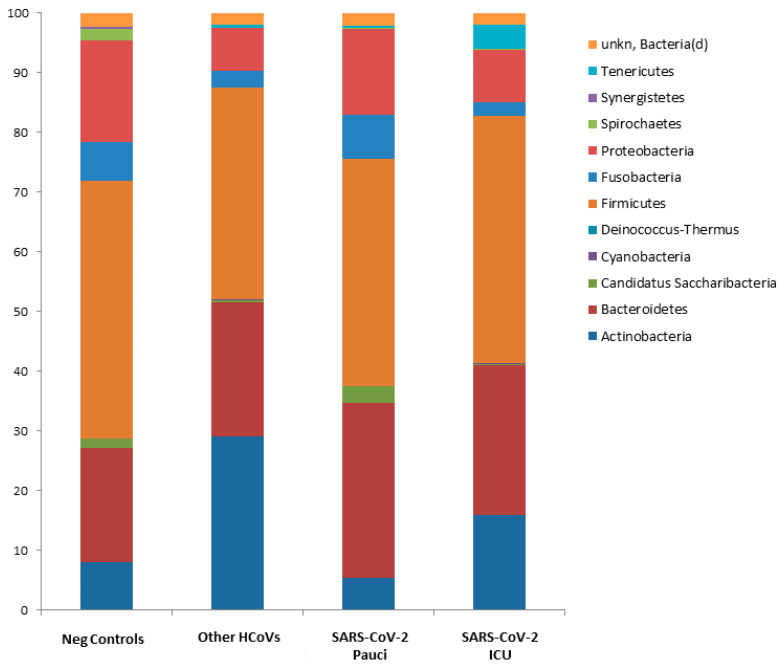
Microbiota composition of Neg Controls, Other HCoVs, SARS-CoV-2 Pauci and SARS-CoV-2 ICU at the phylum level. The mean value of all the detected taxa is represented.

At the phylum level, Deinococcus Thermus was present only in controls as compared to SARS-CoV-2 ICU patients, SARS-CoV-2 Pauci or other HCoVs patients. Candidatus Saccharibacteria (formerly known as TM7) was strongly increased in negative controls and SARS-CoV-2 Pauci patients as compared to SARS-CoV-2 ICU patients and Other HCoVs patients (Figure 2 and Supplementary Table S1).

**Figure 3:**
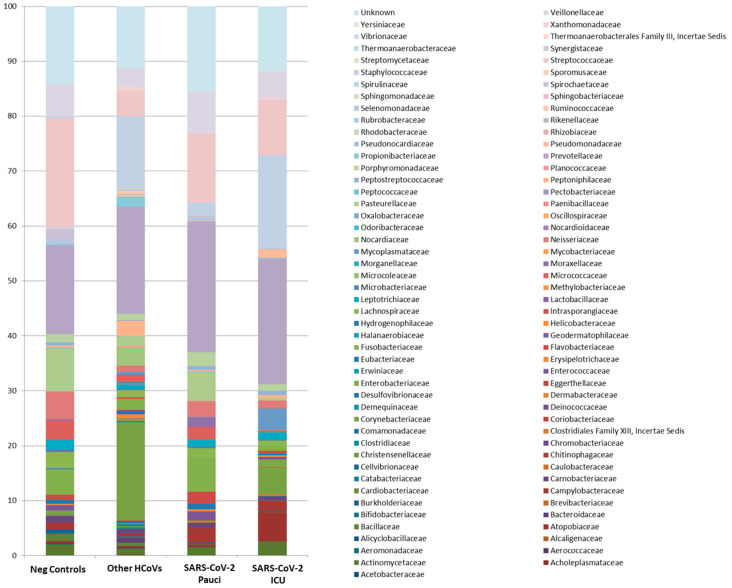
Microbiota composition of Neg Controls, Other HCoVs, SARS-CoV-2 Pauci and SARS-CoV-2 ICU at the family level. The mean value of all the detected taxa is represented.

At the family level, Alicyclobacillaceae, Chromobacteriaceae, Deinococcacaee, Hydrogenophilaceae, Thermoanaerobacteraceae, Sporomusaceae and Thermoanaerobacterales FamilyIII. Incertae Sedis were exclusive microorganisms detected in Neg Control patients, while Pectobacteriaceae were exclusive to SARS-CoV-2 ICU patients (Figure 3 and Supplementary Table S1).

**Figure 4:**
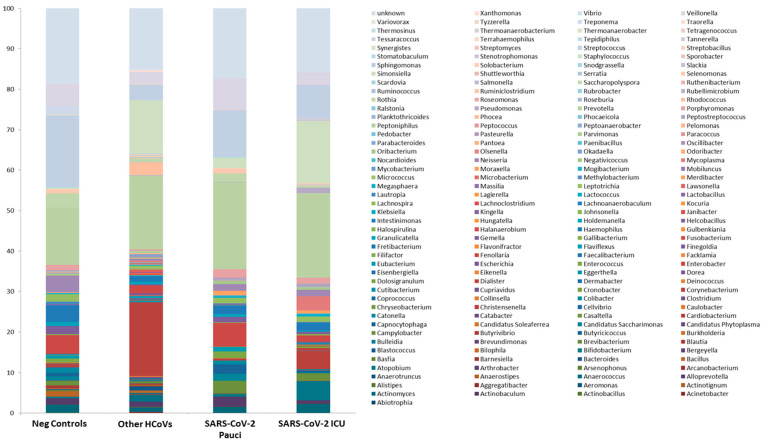
Microbiota composition of Neg Controls, Other HCoVs, SARS-CoV-2 Pauci and SARS-CoV-2 ICU at the genus level. The mean value of all the detected taxa is represented.

At the lower taxonomic level, Johnsonella, Tepidiphilus, Thermoanaerobacter, Thermoanaerobacterium, Thermosinus and Variovorax were exclusive to Neg Control patients, while Salmonella, Scardovia, Serratia and unk_Pseudomonadaceae were included exclusively in SARS-CoV-2 ICU patients (Figure 4 and Supplementary Table S1).

**Figure 5:**
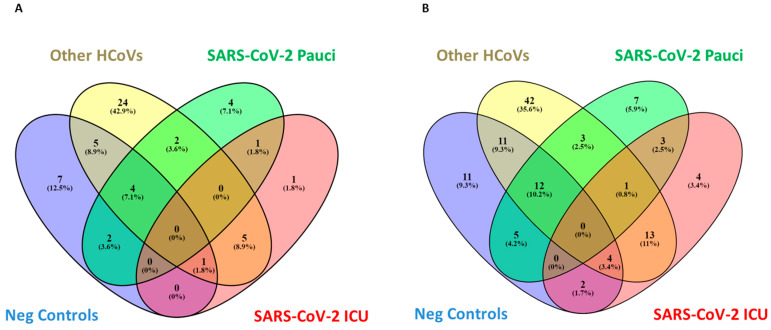
Venn diagrams showing the number of distinct and shared families (**A**) and genera (**B**) up and downregulated between subjects grouped by Neg Controls, other HCoVs, SARS-CoV-2 Pauci and SARS-CoV-2 ICU.

Notably, two microorganisms were detected in all the different groups except for SARS-CoV-2 ICU patients, which resulted in complete depletion of Bifidobacterium and Clostridium, key microorganisms involved in short-chain fatty acid production. Other HCoV-positive patients were characterized by a more abundant richness, as demonstrated by the 24 and 42 families and genera, respectively, detected exclusively in this group of patients (Figure 5A,B and Supplementary Table S1).

### 3.4. Nasal/Ooropharyngeal Microbiota of COVID-19 Patients in Intensive Care Unit as Compared to COVID-19 Paucisymptomatic Patients

Although SARS-CoV-2 Pauci and SARS-CoV-2 ICU patients share three common elements at genus levels, such as *unkn_Campylobacterales, unkn_Clostridiales.Family.XIII_Incertae.Sedis and unkn_Enterococcaceae*, seven elements included exclusively in SARS-CoV-2 Pauci were detected: *Bulleidia, Halanaerobium, Streptobacillus, unkn_Epsilonproteobacteria, unkn_Moraxellaceae, unkn_Mycoplasmataceae, unkn_Tenericutes* and the above-mentioned four genera (*Salmonella, Scardovia, Serratia and unkn_Pseudomonadaceae*) were included exclusively in SARS-CoV-2 ICU patients (Figure 5B, Supplementary Table S1).

## 4. Discussion

In our pilot study, we aimed to investigate the NOP microbiota profiles in patients affected by COVID-19 as compared to non-infected patients and patients infected with a different respiratory virus, such as other HCoVs. In the first instance, both Chao1 and Shannon indexes show a significant decrease in SARS-CoV-2 ICU patients as compared to the other analyzed groups, supporting a strong alteration of the microbiome of the nasopharyngeal tract. This is consistent with studies previously published in the field of other respiratory diseases caused by viral infections [19]. Most studies involving viral infections and the microbiome of the respiratory tract refer to the influenza virus, and similarly, these studies highlight how the infection can disrupt the composition of the bacterial community inhabiting the respiratory tract, especially in the case of severe diseases.

We identified several microorganisms able to differentiate the subjects belonging to the different groups. In particular, we found an increased presence of Deinoccoccus Thermus in Neg Control subjects as compared to SARS-CoV-2 patients or to the HCoVs-positive patients. SARS-CoV-2 ICU patients displayed a complete depletion of Bifidobacterium and Clostridium. The presence of Moraxellacaea spp. was observed exclusively in SARS-CoV-2 Pauci patients, which was associated with stable bacterial community composition and respiratory health [20]. Meanwhile the presence of Pseudomonaceae was found exclusively in SARS-CoV-2 ICU patients, which is known to be associated with pathogenic conditions, in particular with non-influenza-related severe acute respiratory syndromes. In fact, Pseudomonas has been shown to improve mucus production, in which the main component, mucins, seem to have a protective role against influenza virus entry into the cell [19]. Increased Pseudomonas content is also linked to a decrease in the diversity in the microbiome, which often is associated with viral infections [21]. This condition is surely favored by the use of antibiotics, although we have no data regarding the therapy carried out by the study patients. Although we are aware that clinical data such as age and gender are factors that strongly influence the microbiota profiles, in our study population, these demographic features were not statistically significant or different among the three groups. To date, less is known about the microbiome of the respiratory tract’s role in the context of a viral infection and the presence of viral-bacterial interactions has been suggested by epidemiological studies. The biological mechanisms of these bidirectional interactions have been mostly studied for a small slice of viruses and bacteria that are the aetiological agents of respiratory diseases. In fact, respiratory bacteria are thought to promote viral infections through different pathways. Furthermore, the presence of specific bacterial species may regulate the composition of the microbial community and could facilitate the adaptation of the microbiome to novel environments by preserving its diversity. Moreover, a recent study also reported the possible interaction between bacteriophages and the bacterial community diversity in the respiratory tract, which suggests that substantial interaction exists between bacteriophages and the microbiota in the healthy respiratory tract [20]. Therefore, the paths that can be pursued to analyze this aspect of the pathogenesis of COVID-19 are many and intricate, to which the role of host immunity must be added, which is strongly linked to the composition of the resident bacterial community. Our pilot study contains several limitations. Firstly, it is a single center in an urban area in central Italy with a restricted number of enrolled patients. Our preliminary observations on the impact of SARS-CoV-2 infection on NOP microbiota need to be confirmed in a larger cohort including asymptomatic COVID-19 subjects, which could add more information. Nonetheless, the study of the microbiome of the nasopharyngeal tract opens multiple possibilities of the interaction and involvement of this aspect in the development of viral diseases and also of COVID-19.

## 5. Conclusions

The study carried out in this work is a proof of concept and shows that there are differences between individuals infected with different degrees of severity, not infected and infected with other HCoVs. Specific microbial signatures in COVID-19 patients, the role of the gut microbiota in different phases of disease and hospital settings, particularly including therapies, need to be investigated and validated in larger cohorts. The literature is still poor in this regard for COVID-19, but the path of microbiome analysis using 16S rRNA sequencing by Next generation Sequencing NGS could lead to new definitions of prognosis markers and a personalized medicine capable of providing important information about pathogenesis and human–host interaction in the case of the SARS-CoV-2. In conclusion, the data that emerged from this preliminary analysis show that the nasal/oropharyngeal microbiota profiles of SARS-CoV-2 patients admitted to ICU are characterized by a complete depletion of Bifidobacterium and Clostridium, while Salmonella, Scardovia, Serratia and Pectobacteriaceae were exclusively detected in ICU SARS-CoV-2 patients. These data could provide useful information in order to stratify COVID-19 patients and can contribute to the development and research of new intervention strategies that can help improve the outcome of COVID-19 disease.

## Data Availability

The data presented in this study are available on request from the corresponding author.

## Supplementary Materials

The following are available online at https://www.mdpi.com/1660-4601/18/4/2174/s1, Table S1: Comparisons of bacteria mean abundance at different taxa levels among patient groups (only significant results were shown).
