# Supplementary material for: Investigation of Nasal/Oropharyngeal Microbial Community of COVID-19 Patients by 16S rDNA Sequencing
Source: Int J Environ Res Public Health. 2021 Feb 23;18(4):2174. doi: 10.3390/ijerph18042174 (PMC7926517; doi:10.3390/ijerph18042174)
Supplement: Supplementary file 1 [file ijerph-18-02174-s001.pdf]

Supplementary Table 1. Comparisons of bacteria median abundance at phylum (A), Family (B) and Genus (C) among patients groups (only significant results were shown)

A. Phylum

						Group comparisons (p-values)#					
**Bacteria**	**CTRLs neg (N=10)**	**Other HCoVs (N=8)**	**SARS-CoV2 pauci-symptomatic (N=11)**	**SARS-CoV2 ICU (N=10)**	**p-value overall***	**Other HCoVs vs. CTRL**	**Pauci vs. CTRL**	**ICU vs. CTRL**	**Pauci vs. Other HCoVs**	**ICU vs. Other HCoVs**	**ICU vs. Pauci**
Candidatus Saccharibacteria	1.1674 [0.3976 - 2.3924]	0.0125 [0.0065 - 0.3667]	1.9562 [0.6829 - 3.9751]	0.0093 [0.0026 - 0.1047]	0.027	0.062	0.673	0.021	0.078	1.000	0.031
Deinococcus Thermus	0.0071 [0.0032 - 0.0161]	Absent	Absent	Absent	<0.001	0.001	<0.001	<0.001	1.000	1.000	1.000

Median relative abundance (%) along with interquartile range [first-third quartiles]; Absent: all values are 0%
*p-values from Kruskal-Wallis test and were adjusted following the Bonferroni correction for multiple testing
#p-values from Dwass-Steel-Critchlow-Fligner test

B. Family

Bacteria	CTRLs neg (N=10)	Other HCoVs (N=8)	SARS-CoV2 pauci-symptomatic (N=11)	SARS-CoV2 ICU (N=10)	p-value overall*	Group comparisons (p-values)#					
						Other HCoVs vs. CTRL	Pauci vs. CTRL	ICU vs. CTRL	Pauci vs. Other HCoVs	ICU vs. Other HCoVs	ICU vs. Pauci
Acetobacteraceae	Absent	0.0010 [0.0005 - 0.0180]	Absent	Absent	<0.001	<0.001	1.000	1.000	<0.001	<0.001	1.000
Aeromonadaceae	0.0256 [0.0203 - 0.0392]	0.0102 [0.0051 - 0.0374]	0.0325 [0.0138 - 0.0601]	Absent	0.004	0.542	1.000	<0.001	0.550	<0.001	<0.001
Alcaligenaceae	Absent	0.0074 [0.0009 - 0.0215]	Absent	Absent	<0.001	<0.001	1.000	1.000	<0.001	<0.001	1.000
Alicyclobacillaceae	0.0089 [0.0016 - 0.0344]	Absent	Absent	Absent	<0.001	0.001	<0.001	<0.001	1.000	1.000	1.000
Brevibacteriaceae	Absent	0.0257 [0.0009 - 0.1044]	Absent	Absent	<0.001	<0.001	1.000	1.000	<0.001	<0.001	1.000
Caulobacteraceae	0.0060 [0.0033 - 0.0077]	0.0237 [0.0048 - 0.0714]	Absent	Absent	<0.001	0.599	<0.001	<0.001	<0.001	<0.001	1.000
Cellvibrionaceae	Absent	0.0004 [0.0001 - 0.0035]	Absent	Absent	<0.001	0.008	1.000	1.000	0.005	0.008	1.000
Chitinophagaceae	Absent	0.0145 [0.0010 - 0.0271]	Absent	Absent	<0.001	<0.001	1.000	1.000	<0.001	<0.001	1.000
Christensenellaceae	Absent	0.0003 [0.0000 - 0.0009]	Absent	Absent	0.011	0.025	1.000	1.000	0.018	0.025	1.000
Chromobacteriaceae	0.0089 [0.0042 - 0.0366]	Absent	Absent	Absent	<0.001	0.001	<0.001	<0.001	1.000	1.000	1.000
Coriobacteriaceae	Absent	0.0010 [0.0003 - 0.0176]	Absent	Absent	<0.001	0.002	1.000	1.000	0.001	0.002	1.000
Deinococcaceae	0.0053 [0.0020 - 0.0108]	Absent	Absent	Absent	<0.001	0.001	<0.001	<0.001	1.000	1.000	1.000

Demequinaceae	Absent	0.0039 [0.0008 - 0.0127]	Absent	Absent	<0.001	<0.001	1.000	1.000	<0.001	<0.001	1.000
Dermabacteraceae	Absent	0.0019 [0.0014 - 0.0558]	Absent	0.0004 [0.0000 - 0.0014]	<0.001	<0.001	1.000	0.027	<0.001	0.239	0.019
Desulfovibrionaceae	Absent	0.0001 [0.0000 - 0.0004]	Absent	Absent	0.011	0.025	1.000	1.000	0.018	0.025	1.000
Erwiniaceae	Absent	0.0121 [0.0087 - 0.0194]	Absent	0.0032 [0.0010 - 0.0225]	<0.001	<0.001	1.000	0.001	<0.001	0.542	<0.001
Geodermatophilaceae	Absent	0.0064 [0.0004 - 0.0315]	Absent	Absent	<0.001	0.002	1.000	1.000	0.001	0.002	1.000
Halanaerobiaceae	Absent	Absent	0.0103 [0.0054 - 0.0173]	Absent	<0.001	1.000	<0.001	1.000	<0.001	1.000	<0.001
Helicobacteraceae	0.0098 [0.0059 - 0.0128]	Absent	0.0063 [0.0048 - 0.0169]	Absent	<0.001	0.001	0.999	<0.001	<0.001	1.000	<0.001
Hydrogenophilaceae	0.0388 [0.0088 - 0.1464]	Absent	Absent	Absent	<0.001	0.001	<0.001	<0.001	1.000	1.000	1.000
Intrasporangiaceae	0.0078 [0.0043 - 0.0215]	0.0062 [0.0015 - 0.0227]	Absent	Absent	<0.001	0.985	<0.001	<0.001	<0.001	<0.001	1.000
Methylobacteriaceae	Absent	0.0845 [0.0049 - 0.1511]	0.0007 [0.0004 - 0.0012]	Absent	<0.001	0.002	0.003	1.000	0.114	0.002	0.003
Microbacteriaceae	0.0107 [0.0092 - 0.0203]	0.0792 [0.0153 - 0.1389]	Absent	0.0083 [0.0070 - 0.0137]	0.001	0.379	<0.001	0.715	<0.001	0.143	<0.001
Morganellaceae	Absent	0.0152 [0.0060 - 0.0494]	Absent	Absent	<0.001	<0.001	1.000	1.000	<0.001	<0.001	1.000
Mycobacteriaceae	Absent	0.0077 [0.0034 - 0.0274]	Absent	Absent	<0.001	<0.001	1.000	1.000	<0.001	<0.001	1.000
Nocardiaceae	Absent	0.0264 [0.0106 - 0.0693]	Absent	0.0095 [0.0056 - 0.0176]	<0.001	<0.001	1.000	0.001	<0.001	0.285	<0.001
Nocardioidaceae	Absent	0.0120 [0.0028 - 0.0526]	Absent	Absent	<0.001	0.002	1.000	1.000	0.001	0.002	1.000
Odoribacteraceae	Absent	0.0029	Absent	Absent	0.011	0.025	1.000	1.000	0.018	0.025	1.000

		[0.0000 - 0.0086]									
Oxalobacteraceae	0.0115 [0.0042 - 0.0145]	0.0067 [0.0051 - 0.0447]	Absent	Absent	<0.001	0.996	<0.001	<0.001	<0.001	<0.001	1.000
Pectobacteriaceae	Absent	Absent	Absent	0.0009 [0.0002 - 0.0127]	<0.001	1.000	1.000	0.004	1.000	0.010	0.002
Planococcaceae	Absent	0.0061 [0.0024 - 0.0199]	Absent	Absent	<0.001	<0.001	1.000	1.000	<0.001	<0.001	1.000
Pseudonocardiaceae	Absent	0.0083 [0.0023 - 0.0279]	Absent	Absent	<0.001	<0.001	1.000	1.000	<0.001	<0.001	1.000
Rhizobiaceae	Absent	0.0054 [0.0018 - 0.0242]	Absent	Absent	<0.001	<0.001	1.000	1.000	<0.001	<0.001	1.000
Rikenellaceae	Absent	0.0004 [0.0001 - 0.0108]	Absent	Absent	<0.001	0.008	1.000	1.000	0.005	0.008	1.000
Rubrobacteraceae	0.0029 [0.0009 - 0.0118]	0.0186 [0.0003 - 0.0307]	Absent	Absent	<0.001	0.998	<0.001	<0.001	0.001	0.002	1.000
Ruminococcaceae	Absent	0.0078 [0.0018 - 0.0570]	Absent	Absent	<0.001	<0.001	1.000	1.000	<0.001	<0.001	1.000
Sphingobacteriaceae	Absent	0.0110 [0.0024 - 0.0527]	Absent	Absent	<0.001	<0.001	1.000	1.000	<0.001	<0.001	1.000
Sphingomonadaceae	Absent	0.1584 [0.0066 - 0.2531]	Absent	Absent	<0.001	<0.001	1.000	1.000	<0.001	<0.001	1.000
Sporomusaceae	0.0242 [0.0087 - 0.1157]	Absent	Absent	Absent	<0.001	0.001	<0.001	<0.001	1.000	1.000	1.000
Thermoanaerobacteraceae	0.0142 [0.0035 - 0.0848]	Absent	Absent	Absent	<0.001	0.001	<0.001	<0.001	1.000	1.000	1.000
Thermoanaerobacterales Family III Incertae Sedis	0.0167 [0.0047 - 0.0542]	Absent	Absent	Absent	<0.001	0.001	<0.001	<0.001	1.000	1.000	1.000
unknown Actinomycetales	Absent	0.0003 [0.0001 - 0.0115]	Absent	Absent	<0.001	0.008	1.000	1.000	0.005	0.008	1.000
unknown Alphaproteobacteria	Absent	0.0120 [0.0081 - 0.0300]	0.0090 [0.0059 - 0.0109]	Absent	<0.001	<0.001	<0.001	1.000	0.755	<0.001	<0.001

unknown Burkholderiales	0.0140 [0.0068 - 0.0145]	0.0263 [0.0055 - 0.0374]	Absent	Absent	<0.001	0.925	<0.001	<0.001	<0.001	<0.001	1.000
unknown Campylobacterales	Absent	Absent	0.0138 [0.0093 - 0.0237]	0.0060 [0.0020 - 0.0103]	<0.001	1.000	<0.001	<0.001	<0.001	0.001	0.259
unknown Epsilonproteobacteria	Absent	Absent	0.0071 [0.0044 - 0.0185]	Absent	<0.001	1.000	<0.001	1.000	<0.001	1.000	<0.001
unknown Erysipelotrichales	Absent	0.0005 [0.0002 - 0.0071]	Absent	Absent	<0.001	0.008	1.000	1.000	0.005	0.008	1.000
unknown Erysipelotrichia	Absent	0.0011 [0.0000 - 0.0041]	Absent	Absent	0.011	0.025	1.000	1.000	0.018	0.025	1.000
unknown Flavobacteriales	Absent	Absent	0.0091 [0.0040 - 0.0130]	Absent	<0.001	1.000	<0.001	1.000	0.003	1.000	<0.001
unknown Fusobacteriales	0.0134 [0.0070 - 0.0267]	0.0002 [0.0000 - 0.0062]	0.0149 [0.0104 - 0.0278]	Absent	0.016	0.115	0.992	<0.001	0.298	0.070	<0.001
unknown Mollicutes	0.0080 [0.0016 - 0.0397]	Absent	0.0119 [0.0059 - 0.0742]	Absent	<0.001	0.001	0.494	<0.001	<0.001	1.000	<0.001
unknown Neisseriales	0.0129 [0.0044 - 0.0708]	0.0048 [0.0006 - 0.0158]	0.0031 [0.0005 - 0.0117]	Absent	0.009	0.542	0.259	<0.001	1.000	0.002	<0.001
unknown Tenericutes	Absent	Absent	0.0070 [0.0047 - 0.0233]	Absent	<0.001	1.000	<0.001	1.000	<0.001	1.000	<0.001
unknown Tissierellia	0.0136 [0.0035 - 0.0296]	0.0076 [0.0026 - 0.0241]	0.0066 [0.0043 - 0.0339]	Absent	0.007	0.939	1.000	<0.001	0.988	<0.001	<0.001
Xanthomonadaceae	Absent	0.0272 [0.0028 - 0.0511]	Absent	0.0053 [0.0029 - 0.0133]	<0.001	<0.001	1.000	<0.001	<0.001	0.855	<0.001
Yersiniaceae	Absent	0.0078 [0.0048 - 0.0155]	Absent	0.0018 [0.0013 - 0.0155]	<0.001	<0.001	1.000	<0.001	<0.001	0.951	<0.001

Median relative abundance (%) along with interquartile range [first-third quartiles]; Absent: all values are 0%

*p-values from Kruskal-Wallis test and were adjusted following the Bonferroni correction for multiple testing

#p-values from Dwass-Steel-Critchlow-Fligner test

C. Genus

Bacteria	CTRLs neg (N=10)	Other HCoVs (N=8)	SARS-CoV2 pauci-symptomatic (N=11)	SARS-CoV2 ICU (N=10)	p-value overall*	Group comparisons (p-values)#					
						Other HCoVs vs. CTRL	Pauci vs. CTRL	ICU vs. CTRL	Pauci vs. Other HCoVs	ICU vs. Other HCoVs	ICU vs. Pauci
Acinetobacter	0.0142 [0.0051 - 0.0259]	0.0494 [0.0065 - 0.1936]	0.0028 [0.0011 - 0.0264]	Absent	0.013	0.811	0.833	<0.001	0.350	<0.001	<0.001
Aeromonas	0.0257 [0.0201 - 0.0391]	0.0093 [0.0038 - 0.0371]	0.0320 [0.0137 - 0.0593]	Absent	0.008	0.542	1.000	<0.001	0.497	<0.001	<0.001
Alistipes	Absent	0.0002 [0.0001 - 0.0080]	Absent	Absent	0.002	0.008	1.000	1.000	0.005	0.008	1.000
Anaerostipes	Absent	0.0030 [0.0007 - 0.0126]	Absent	Absent	0.002	0.008	1.000	1.000	0.005	0.008	1.000
Arsenophonus	Absent	0.0055 [0.0021 - 0.0188]	Absent	Absent	<0.001	0.002	1.000	1.000	0.001	0.002	1.000
Arthrobacter	0.0124 [0.0045 - 0.0180]	0.0373 [0.0150 - 0.0548]	Absent	Absent	<0.001	0.243	<0.001	<0.001	<0.001	<0.001	1.000
Bergeyella	0.0799 [0.0311 - 0.0950]	0.0002 [0.0000 - 0.0137]	0.0477 [0.0242 - 0.0896]	Absent	0.004	0.013	0.866	<0.001	0.045	0.025	<0.001
Bifidobacterium	0.0079 [0.0009 - 0.0413]	0.0084 [0.0017 - 0.0236]	0.0014 [0.0003 - 0.0061]	Absent	0.033	0.998	0.538	<0.001	0.496	<0.001	0.007
Blastococcus	Absent	0.0033 [0.0001 - 0.0130]	Absent	Absent	0.002	0.008	1.000	1.000	0.005	0.008	1.000
Blautia	Absent	0.0002 [0.0001 - 0.0153]	Absent	Absent	0.002	0.008	1.000	1.000	0.005	0.008	1.000
Brevibacterium	Absent	0.0256 [0.0009 - 0.1055]	Absent	Absent	<0.001	<0.001	1.000	1.000	<0.001	<0.001	1.000
Brevundimonas	Absent	0.0105 [0.0024 - 0.0375]	Absent	Absent	<0.001	<0.001	1.000	1.000	<0.001	<0.001	1.000

Bulleidia	Absent	Absent	0.0023 [0.0004 - 0.0156]	Absent	0.005	1.000	0.007	1.000	0.017	1.000	0.007
Burkholderia	0.0255 [0.0101 - 0.1044]	0.0024 [0.0017 - 0.0031]	Absent	Absent	<0.001	0.023	<0.001	<0.001	<0.001	<0.001	1.000
Butyrivibrio	0.0085 [0.0039 - 0.0469]	Absent	0.0108 [0.0030 - 0.0159]	Absent	<0.001	0.001	0.961	<0.001	0.003	1.000	<0.001
Casaltella	Absent	0.0007 [0.0001 - 0.0339]	Absent	Absent	0.002	0.008	1.000	1.000	0.005	0.008	1.000
Caulobacter	Absent	0.0055 [0.0002 - 0.0230]	Absent	Absent	<0.001	0.002	1.000	1.000	0.001	0.002	1.000
Chryseobacterium	Absent	0.0085 [0.0040 - 0.0139]	Absent	Absent	<0.001	0.002	1.000	1.000	0.001	0.002	1.000
Clostridium	0.0477 [0.0140 - 0.0743]	0.0417 [0.0098 - 0.1045]	0.0221 [0.0146 - 0.0486]	Absent	0.014	1.000	0.943	<0.001	0.976	<0.001	<0.001
Cupriavidus	Absent	0.0025 [0.0003 - 0.0154]	Absent	Absent	0.002	0.008	1.000	1.000	0.005	0.008	1.000
Deinococcus	0.0046 [0.0013 - 0.0109]	Absent	Absent	Absent	<0.001	0.001	<0.001	<0.001	1.000	1.000	1.000
Dorea	Absent	0.0004 [0.0000 - 0.0055]	Absent	Absent	0.021	0.025	1.000	1.000	0.018	0.025	1.000
Enterobacter	Absent	0.0160 [0.0109 - 0.0548]	Absent	0.0023 [0.0014 - 0.0539]	<0.001	<0.001	1.000	0.001	<0.001	0.542	<0.001
Escherichia	Absent	0.0134 [0.0035 - 0.0191]	Absent	0.0112 [0.0030 - 0.0398]	<0.001	<0.001	1.000	0.001	<0.001	1.000	<0.001
Facklamia	Absent	0.0001 [0.0001 - 0.0162]	Absent	Absent	0.002	0.008	1.000	1.000	0.005	0.008	1.000
Filifactor	0.0452 [0.0057 - 0.1881]	0.0023 [0.0003 - 0.0092]	0.0211 [0.0104 - 0.1108]	Absent	0.032	0.204	0.980	0.001	0.164	0.008	<0.001
Gulbenkiania	0.0083 [0.0014 - 0.0328]	Absent	Absent	Absent	<0.001	0.004	<0.001	0.001	1.000	1.000	1.000
Halanaerobium	Absent	Absent	0.0104	Absent	<0.001	1.000	<0.001	1.000	<0.001	1.000	<0.001

			[0.0053 - 0.0171]								
Holdemanella	Absent	0.0002 [0.0000 - 0.0050]	Absent	Absent	0.021	0.025	1.000	1.000	0.018	0.025	1.000
Janibacter	Absent	0.0006 [0.0001 - 0.0043]	Absent	Absent	0.002	0.008	1.000	1.000	0.005	0.008	1.000
Johnsonella	0.0029 [0.0004 - 0.0126]	Absent	Absent	Absent	<0.001	0.004	<0.001	0.001	1.000	1.000	1.000
Klebsiella	Absent	0.0071 [0.0034 - 0.0195]	Absent	0.0028 [0.0007 - 0.0390]	<0.001	<0.001	1.000	<0.001	<0.001	0.763	<0.001
Kocuria	0.0074 [0.0046 - 0.0120]	0.0099 [0.0028 - 0.0618]	Absent	0.0037 [0.0018 - 0.0065]	0.003	0.993	<0.001	0.304	<0.001	0.486	<0.001
Lachnoclostridium	Absent	0.0030 [0.0000 - 0.0129]	Absent	0.0018 [0.0004 - 0.0036]	0.008	0.025	1.000	0.001	0.018	0.999	<0.001
Lactococcus	0.0112 [0.0088 - 0.0124]	Absent	Absent	0.0082 [0.0023 - 0.0145]	<0.001	0.001	<0.001	0.759	1.000	0.004	<0.001
Lawsonella	Absent	0.0384 [0.0120 - 0.2404]	Absent	0.0025 [0.0001 - 0.0073]	0.004	0.002	1.000	0.010	0.001	0.349	0.007
Massilia	Absent	0.0033 [0.0024 - 0.0239]	Absent	Absent	<0.001	<0.001	1.000	1.000	<0.001	<0.001	1.000
Methylobacterium	Absent	0.0852 [0.0049 - 0.1521]	0.0005 [0.0003 - 0.0011]	Absent	0.001	0.002	0.003	1.000	0.114	0.002	0.003
Microbacterium	0.0080 [0.0050 - 0.0142]	0.0516 [0.0128 - 0.0709]	Absent	Absent	<0.001	0.379	<0.001	<0.001	<0.001	<0.001	1.000
Micrococcus	Absent	0.0112 [0.0052 - 0.0298]	Absent	0.0044 [0.0020 - 0.0084]	<0.001	<0.001	1.000	0.001	<0.001	0.542	<0.001
Mycobacterium	Absent	0.0072 [0.0032 - 0.0256]	Absent	Absent	<0.001	<0.001	1.000	1.000	<0.001	<0.001	1.000
Negativicoccus	Absent	0.0037 [0.0001 - 0.0119]	Absent	Absent	0.002	0.008	1.000	1.000	0.005	0.008	1.000
Nocardioides	Absent	0.0111 [0.0020 - 0.0324]	Absent	Absent	0.002	0.008	1.000	1.000	0.005	0.008	1.000

Odoribacter	Absent	0.0001 [0.0000 - 0.0013]	Absent	Absent	0.021	0.025	1.000	1.000	0.018	0.025	1.000
Okadaella	0.0023 [0.0006 - 0.0056]	Absent	Absent	0.0003 [0.0000 - 0.0016]	0.009	0.004	<0.001	0.379	1.000	0.054	0.019
Olsenella	Absent	0.0020 [0.0000 - 0.0078]	Absent	0.0093 [0.0048 - 0.0192]	<0.001	0.025	1.000	<0.001	0.018	0.241	<0.001
Paenibacillus	Absent	0.0106 [0.0073 - 0.0119]	Absent	0.0077 [0.0049 - 0.0126]	<0.001	<0.001	1.000	<0.001	<0.001	0.998	<0.001
Pantoea	Absent	0.0069 [0.0046 - 0.0105]	Absent	0.0014 [0.0002 - 0.0182]	<0.001	<0.001	1.000	0.004	<0.001	0.599	0.002
Parabacteroides	Absent	0.0002 [0.0001 - 0.0101]	Absent	Absent	0.002	0.008	1.000	1.000	0.005	0.008	1.000
Paracoccus	Absent	0.0039 [0.0002 - 0.0286]	Absent	Absent	<0.001	0.002	1.000	1.000	0.001	0.002	1.000
Pasteurella	0.0020 [0.0010 - 0.0109]	Absent	0.0092 [0.0025 - 0.0146]	Absent	0.005	0.004	0.961	0.001	0.007	1.000	0.003
Pedobacter	Absent	0.0072 [0.0013 - 0.0188]	Absent	Absent	<0.001	<0.001	1.000	1.000	<0.001	<0.001	1.000
Pelomonas	0.0093 [0.0018 - 0.0206]	0.1057 [0.0150 - 0.2862]	Absent	Absent	<0.001	0.379	<0.001	0.001	<0.001	<0.001	1.000
Ralstonia	0.0055 [0.0006 - 0.0112]	0.0309 [0.0060 - 0.0958]	Absent	Absent	<0.001	0.811	<0.001	<0.001	0.001	0.002	1.000
Rhodococcus	Absent	0.0250 [0.0100 - 0.0635]	Absent	0.0069 [0.0053 - 0.0161]	<0.001	<0.001	1.000	0.001	<0.001	0.206	<0.001
Roseburia	Absent	0.0001 [0.0000 - 0.0033]	Absent	Absent	0.021	0.025	1.000	1.000	0.018	0.025	1.000
Rubrobacter	0.0029 [0.0009 - 0.0119]	0.0187 [0.0002 - 0.0309]	Absent	Absent	<0.001	0.998	<0.001	<0.001	0.001	0.002	1.000
Ruminiclostridium	Absent	0.0001 [0.0000 - 0.0003]	Absent	Absent	0.021	0.025	1.000	1.000	0.018	0.025	1.000
Ruminococcus	Absent	0.0010	Absent	Absent	<0.001	0.002	1.000	1.000	0.001	0.002	1.000

		[0.0006 - 0.0199]									
Saccharopolyspora	Absent	0.0002 [0.0001 - 0.0028]	Absent	Absent	<0.001	0.002	1.000	1.000	0.001	0.002	1.000
Salmonella	Absent	Absent	Absent	0.0013 [0.0001 - 0.0106]	0.007	1.000	1.000	0.010	1.000	0.024	0.007
Scardovia	Absent	Absent	Absent	0.0002 [0.0000 - 0.0014]	0.046	1.000	1.000	0.027	1.000	0.054	0.019
Serratia	Absent	Absent	Absent	0.0016 [0.0013 - 0.0088]	<0.001	1.000	1.000	<0.001	1.000	0.001	<0.001
Snodgrassella	0.0028 [0.0004 - 0.0135]	0.0116 [0.0000 - 0.1746]	Absent	Absent	0.027	0.970	0.002	0.004	0.018	0.025	1.000
Sphingomonas	Absent	0.1386 [0.0050 - 0.2163]	Absent	Absent	<0.001	<0.001	1.000	1.000	<0.001	<0.001	1.000
Stenotrophomonas	Absent	0.0021 [0.0010 - 0.0211]	Absent	0.0022 [0.0012 - 0.0062]	<0.001	<0.001	1.000	<0.001	<0.001	0.993	<0.001
Streptobacillus	Absent	Absent	0.0002 [0.0000 - 0.0300]	Absent	0.028	1.000	0.018	1.000	0.038	1.000	0.018
Tepidiphilus	0.0360 [0.0085 - 0.1425]	Absent	Absent	Absent	<0.001	0.001	<0.001	<0.001	1.000	1.000	1.000
Thermoanaerobacter	0.0139 [0.0033 - 0.0839]	Absent	Absent	Absent	<0.001	0.001	<0.001	<0.001	1.000	1.000	1.000
Thermoanaerobacterium	0.0161 [0.0043 - 0.0532]	Absent	Absent	Absent	<0.001	0.001	<0.001	<0.001	1.000	1.000	1.000
Thermosinus	0.0177 [0.0059 - 0.0932]	Absent	Absent	Absent	<0.001	0.001	<0.001	<0.001	1.000	1.000	1.000
Tyzzerella	Absent	0.0009 [0.0000 - 0.0080]	Absent	Absent	0.021	0.025	1.000	1.000	0.018	0.025	1.000
unknown Actinomycetales	Absent	0.0003 [0.0001 - 0.0116]	Absent	Absent	0.002	0.008	1.000	1.000	0.005	0.008	1.000
unknown Alicyclobacillaceae	0.0055 [0.0011 - 0.0210]	Absent	Absent	Absent	<0.001	0.001	<0.001	<0.001	1.000	1.000	1.000

unknown Alphaproteobacteria	Absent	0.0122 [0.0081 - 0.0304]	0.0091 [0.0059 - 0.0109]	Absent	<0.001	<0.001	<0.001	1.000	0.706	<0.001	<0.001
unknown Bacillaceae	0.0115 [0.0093 - 0.0367]	0.0120 [0.0074 - 0.0169]	Absent	Absent	<0.001	0.971	<0.001	<0.001	<0.001	<0.001	1.000
unknown Bifidobacteriaceae	0.0014 [0.0008 - 0.0040]	Absent	0.0008 [0.0001 - 0.0014]	Absent	0.023	0.010	0.712	0.004	0.017	1.000	0.007
unknown Burkholderiaceae	0.0069 [0.0032 - 0.0382]	0.0070 [0.0010 - 0.0415]	Absent	Absent	<0.001	0.855	<0.001	<0.001	<0.001	<0.001	1.000
unknown Burkholderiales	0.0081 [0.0048 - 0.0121]	0.0147 [0.0033 - 0.0247]	Absent	Absent	<0.001	0.855	<0.001	<0.001	<0.001	<0.001	1.000
unknown Campylobacterales	Absent	Absent	0.0138 [0.0093 - 0.0237]	0.0060 [0.0020 - 0.0103]	<0.001	1.000	<0.001	<0.001	<0.001	0.001	0.259
unknown Clostridiaceae	0.0076 [0.0068 - 0.0114]	0.0039 [0.0013 - 0.0104]	Absent	Absent	<0.001	0.431	<0.001	<0.001	<0.001	<0.001	1.000
unknown Clostridiales Family XIII Incertae Sedis	Absent	Absent	0.0073 [0.0046 - 0.0229]	0.0011 [0.0000 - 0.0048]	0.005	1.000	<0.001	0.010	0.003	0.024	0.385
unknown Corynebacteriaceae	Absent	0.0074 [0.0011 - 0.0134]	Absent	0.0058 [0.0010 - 0.0218]	<0.001	<0.001	1.000	0.001	<0.001	1.000	<0.001
unknown Corynebacteriales	0.0129 [0.0086 - 0.0216]	0.0462 [0.0205 - 0.1855]	Absent	0.0172 [0.0034 - 0.0386]	0.006	0.379	<0.001	1.000	<0.001	0.379	<0.001
unknown Dermabacteraceae	Absent	0.0007 [0.0002 - 0.0325]	Absent	Absent	<0.001	0.002	1.000	1.000	0.001	0.002	1.000
unknown Enterobacteriaceae	0.0053 [0.0029 - 0.0130]	0.0956 [0.0152 - 0.3191]	Absent	0.0087 [0.0047 - 0.9212]	0.002	0.049	<0.001	0.573	<0.001	0.893	<0.001
unknown Enterococcaceae	Absent	Absent	0.0015 [0.0008 - 0.0024]	0.0013 [0.0010 - 0.1253]	<0.001	1.000	<0.001	<0.001	<0.001	0.001	0.992
unknown Epsilonproteobacteria	Absent	Absent	0.0072 [0.0045 - 0.0185]	Absent	<0.001	1.000	<0.001	1.000	<0.001	1.000	<0.001
unknown Erysipelotrichaceae	Absent	0.0014 [0.0003 - 0.0033]	Absent	Absent	0.002	0.008	1.000	1.000	0.005	0.008	1.000

unknown Erysipelotrichales	Absent	0.0005 [0.0002 - 0.0072]	Absent	Absent	0.002	0.008	1.000	1.000	0.005	0.008	1.000
unknown Erysipelotrichia	Absent	0.0011 [0.0000 - 0.0041]	Absent	Absent	0.021	0.025	1.000	1.000	0.018	0.025	1.000
unknown Eubacteriaceae	Absent	0.0053 [0.0005 - 0.0151]	0.0124 [0.0054 - 0.0154]	Absent	<0.001	0.002	<0.001	1.000	0.862	0.002	<0.001
unknown Flavobacteriaceae	0.0084 [0.0051 - 0.0198]	0.0150 [0.0029 - 0.0197]	0.0083 [0.0051 - 0.0478]	Absent	0.025	1.000	0.961	<0.001	0.880	0.002	<0.001
unknown Fusobacteriaceae	0.0062 [0.0032 - 0.0136]	0.0000 [0.0000 - 0.0070]	0.0138 [0.0041 - 0.0401]	Absent	0.042	0.198	0.921	<0.001	0.234	0.170	<0.001
unknown Fusobacteriales	0.0134 [0.0071 - 0.0269]	0.0002 [0.0000 - 0.0063]	0.0150 [0.0104 - 0.0279]	Absent	0.031	0.115	0.992	<0.001	0.298	0.070	<0.001
unknown Intrasporangiaceae	Absent	0.0005 [0.0002 - 0.0063]	Absent	Absent	<0.001	0.002	1.000	1.000	0.001	0.002	1.000
unknown Microbacteriaceae	Absent	0.0096 [0.0017 - 0.0188]	Absent	Absent	<0.001	<0.001	1.000	1.000	<0.001	<0.001	1.000
unknown Micrococcaceae	0.0645 [0.0326 - 0.1172]	0.0295 [0.0061 - 0.0574]	0.0491 [0.0192 - 0.1137]	Absent	0.019	0.486	0.943	<0.001	0.912	<0.001	<0.001
unknown Mollicutes	0.0079 [0.0016 - 0.0398]	Absent	0.0116 [0.0059 - 0.0745]	Absent	<0.001	0.001	0.494	<0.001	<0.001	1.000	<0.001
unknown Moraxellaceae	Absent	Absent	0.0004 [0.0002 - 0.0039]	Absent	<0.001	1.000	<0.001	1.000	0.003	1.000	<0.001
unknown Mycoplasmataceae	Absent	Absent	0.0123 [0.0066 - 0.0192]	Absent	<0.001	1.000	<0.001	1.000	<0.001	1.000	<0.001
unknown Neisseriales	0.0130 [0.0044 - 0.0711]	0.0048 [0.0006 - 0.0160]	0.0031 [0.0005 - 0.0117]	Absent	0.018	0.542	0.259	<0.001	1.000	0.002	<0.001
unknown Nocardiaceae	Absent	0.0008 [0.0003 - 0.0032]	Absent	Absent	<0.001	0.002	1.000	1.000	0.001	0.002	1.000
unknown Peptoniphilaceae	Absent	0.0049 [0.0017 - 0.0144]	Absent	Absent	<0.001	<0.001	1.000	1.000	<0.001	<0.001	1.000
unknown	0.0094	Absent	0.0099	Absent	<0.001	0.001	0.992	<0.001	0.003	1.000	<0.001

Porphyromonadaceae	[0.0034 - 0.0204]		[0.0089 - 0.0221]								
unknown Propionibacteriaceae	0.0028 [0.0015 - 0.0093]	0.1352 [0.0262 - 1.7542]	Absent	0.0080 [0.0006 - 0.0839]	0.009	0.243	<0.001	0.990	<0.001	0.330	0.002
unknown Pseudomonadaceae	Absent	Absent	Absent	0.0013 [0.0008 - 0.0017]	<0.001	1.000	1.000	<0.001	1.000	0.001	<0.001
unknown Rhodobacteraceae	Absent	0.0055 [0.0019 - 0.0369]	Absent	Absent	<0.001	0.002	1.000	1.000	0.001	0.002	1.000
unknown Ruminococcaceae	Absent	0.0019 [0.0008 - 0.0049]	Absent	Absent	<0.001	0.002	1.000	1.000	0.001	0.002	1.000
unknown Selenomonadaceae	0.0051 [0.0037 - 0.0191]	Absent	Absent	Absent	<0.001	0.001	<0.001	<0.001	1.000	1.000	1.000
unknown Sphingomonadaceae	Absent	0.0106 [0.0015 - 0.0311]	Absent	Absent	<0.001	0.002	1.000	1.000	0.001	0.002	1.000
unknown Sporomusaceae	0.0057 [0.0012 - 0.0208]	Absent	Absent	Absent	0.001	0.010	0.002	0.004	1.000	1.000	1.000
unknown Staphylococcaceae	Absent	0.0053 [0.0032 - 0.0131]	0.0044 [0.0031 - 0.0053]	0.0069 [0.0013 - 0.0508]	0.015	<0.001	<0.001	<0.001	0.912	1.000	0.999
unknown Tenericutes	Absent	Absent	0.0070 [0.0047 - 0.0234]	Absent	<0.001	1.000	<0.001	1.000	<0.001	1.000	<0.001
unknown Tissierellia	0.0047 [0.0012 - 0.0274]	0.0028 [0.0011 - 0.0053]	0.0033 [0.0012 - 0.0280]	Absent	0.016	0.990	1.000	<0.001	1.000	<0.001	<0.001
unknown Xanthomonadaceae	Absent	0.0019 [0.0005 - 0.0193]	Absent	0.0011 [0.0003 - 0.0022]	<0.001	<0.001	1.000	0.001	<0.001	0.763	<0.001
Variovorax	0.0030 [0.0016 - 0.0085]	Absent	Absent	Absent	<0.001	0.001	<0.001	<0.001	1.000	1.000	1.000
Xanthomonas	Absent	0.0020 [0.0005 - 0.0103]	Absent	Absent	<0.001	0.002	1.000	1.000	0.001	0.002	1.000

Median relative abundance (%) along with interquartile range [first-third quartiles]; Absent: all values are 0%

*p-values from Kruskal-Wallis test and were adjusted following the Bonferroni correction for multiple testing

#p-values from Dwass-Steel-Critchlow-Fligner test
